# Is this Judaism? The Question of the Consistency of Israeli Policy and Actions in Gaza with Jewish Thought and Ethics: Part 1: Formulating the problem and methodological and hermeneutic considerations

**DOI:** 10.1007/s11673-025-10489-6

**Published:** 2025-08-04

**Authors:** Paul A. Komesaroff, Jeremiah Z. Kenner

**Affiliations:** 1 Monash University, Melbourne, VIC Australia; 2 The National Health and Medical Research Council of Australia, Canberra, Australia; 3 Melbourne, Australia

**Keywords:** Ethics, War, Conflict, Judaism, Talmud, War crimes, Human shields, Self-defence, Bible, Religion, Collective punishment, Israel, Gaza, Palestine, IDF, Hamas, Geopolitical ethics

## Abstract

There has been much discussion about the tactics used by the Israeli Defence Forces (IDF) and government in the conflict in Gaza following October 7, 2023, which have caused, among other things, systematic destruction of hospitals and schools, the deaths of large numbers of civilians, including women and children, mass starvation, and denial of humanitarian aid. The Israeli government and IDF have sought to justify their actions using ethical arguments, many of which relate to their proclaimed role as the representatives of the Jewish state and of Jewish culture and history. Arguing from the extensive corpus of Jewish ethical thought, extending back thousands of years, this article poses a simple question: Are the above actions by the Israeli government and IDF in Gaza consistent with the ethical tradition of Judaism and the obligations that flow from it? To answer this question, key texts are analysed, especially the Hebrew Bible and the Talmud, and multiple arguments are examined, taking into account the complexities of context and diverse interpretive theories. The paper is presented in two parts, the first discussing the question and methodological issues and the second providing the data and conclusions. We conclude that the alleged acts of the Israeli government and IDF in Gaza are clearly and directly contrary to the Judaic tradition of ethics as it has developed over the millennia. The conduct of the war cannot truthfully be presented in any meaningful sense as representing, or indeed, consistent with, Jewish culture or ethics. These findings have potentially far-reaching consequences, including for the claimed status of Israel as a Jewish state, the relationship between criticism of the government of Israel and the scourge of antisemitism, and the identity of Jewish people both within and outside Israel.

## Introduction

This essay explores a single question: do authoritative texts and ethical traditions that are demonstrably “Jewish” support the current policy of the Israeli government and the conduct of the Israeli Defence Force (IDF) in Gaza in the period from October 7, 2023 to the present?

This question is often discussed in the context of a series of related questions. These cover multiple issues, including: the history and current political status of Gaza and the Palestinian independence movement, the relevance of understanding Israel as a democratic or Jewish state or both, the degree to which criticism of the Israeli government and its policies can be attributed to antisemitism (including debates over competing definitions of this term), and international law and ethical frameworks used to define the criteria for and boundaries of a “just war.” Although each of these additional issues is, independently worthy of extended discussion they will not be directly examined in this essay. Instead, we will focus on the question of consistency with Judaic thought and, in particular, ethics. Despite this narrow focus many of our conclusions may have a bearing on related debates about the justifiability of the Israeli actions in Gaza and the relationship between the Israeli State and “the Jewish people” in general or Jewish individuals living outside Israel in particular.

The essay will proceed in two parts. In part 1, after a brief description of the factual context the specific problem to be addressed will be defined, followed by a description of the methodology to be applied relating to the interpretation of various texts in the Judaic tradition. Part 2 will then present the data obtained from this analysis, together with its conclusions. Specifically, a discussion will be undertaken of passages in the Bible and the subsequent rabbinic literature, including especially the Talmud, which bear on an ethical analysis of the actions of the Israeli army in Gaza. Conclusions will be drawn about whether these actions are consistent with, or contrary to, the ethical imperatives that emerge from the corpus of reflection, insight, and wisdom that has accumulated over the millennia. Because the issues discussed are potentially so consequential we have chosen to present the evidence in a comprehensive manner in order to facilitate further analysis and commentary.

## The Factual Background

On October 7, 2023, armed fighters identified with Hamas, a political entity with control of Gaza, and other aligned Palestinian militant groups (hereinafter “Hamas”), entered Israeli territory contiguous to Gaza and proceeded to kill approximately 1200 Israelis of all ages, including civilians and members of the IDF assigned to military outposts close to the Gaza–Israel border who were not engaged in active combat on that morning. The Hamas operatives took roughly 250 hostages, of whom some have since died in custody and some have been released as part of ceasefire agreements.

The Hamas attack was unprecedented in its tactical scope, ferocity, and success. The loss of life on the Israeli side constituted the greatest number of Jewish lives lost on a single day since the Second World War. The impact of the attack on Israeli Jews and Jews in the diaspora has been profound, including well-documented trauma that is ongoing, and it has re-shaped or reinforced Israeli and diaspora Jewish sensibilities and global attitudes toward Israel.

After a slight pause, the Israeli response to the attack has been massive and all-encompassing, leading to widespread destruction and devastation, despite high levels of organization and publicly advertised strategic objectives. It has involved extensive operations across the whole of Gaza which have caused multiple relocations of large numbers of the Gazan population, the deaths of tens of thousands of civilians—at the time of writing, estimates range from 56,000 to 186,000—including a very large number of women and children, the destruction of infrastructure, hospitals, schools, and houses, and mass starvation and the spread of disease.

There is overwhelming and irrefutable evidence that actions carried out on behalf, and on the instructions, of the Israeli government have also included, among other things: torture of prisoners, sexual violence, imprisonment without trial or due legal process, illegal appropriation of property and destruction of livelihoods, attacks on hospitals and health workers, murder of humanitarian workers, destruction of places of learning and worship, murder of journalists, use of human shields, prevention of delivery of food and other humanitarian aid, and other examples of refusal to abide by the requirements of international law. Evidence of these practices has been documented by both international and Israeli sources—including B’Tselem, Amnesty International, Physicians for Human Rights Israel, Standing Together and many others (Amnesty International 2024; Anonymous 2025; B’Tselem 2004; Human Rights Watch 2025; Physicians for Human Rights 2025; United Nations 2024a, 2024b).^1^

The extent and ferocity of the Israeli response has attracted widespread international condemnation and has raised questions about its underlying moral justification. This is the key concern of this essay.

## The Key Question

It is assumed in this article that Israel as a state, and the government of Israel as its representative, consider themselves to be acting in accordance with the principles of Judaism, as represented in the Jewish literature and traditions. This assumption is grounded in the fact that claims to justify the above acts have been made in arguments presented by the government, its allies, and its media mouthpieces that explicitly or implicitly reference Jewish texts and claim that Israel’s actions are in accordance with ethical and legal principles of Judaism. As a consequence, there has been unprecedented discussion globally about the nature and role of the Israeli state and, in particular, its relationship with the history, culture, and ethical traditions of the Jewish people.

The claim by those making the decisions in Israel that their actions are justified by Jewish law and ethics is the key issue examined in this article. Specifically, we analyse in detail the following question: *Are the acts of the Israeli government and the IDF consistent with the philosophy and ethics of Judaism?* In particular, we examine whether the Judaic tradition permits, supports, or is consistent with the specific actions and outcomes listed above, including attacks on non-combatant civilians, collective punishment, and the use and the targeting of human shields.

The question of whether the Israeli government is acting in accordance with, and in support of, the traditions and interests of Jews and Judaism has significant implications for the lives and well-being of many individuals, Jewish and non-Jewish, and for international relations. It also refers to a larger theoretical question that has arisen in diverse contexts: the legitimacy of a group of individuals who have obtained control, by democratic or other means, over a formidable military apparatus, to speak on behalf of a cultural and ethical tradition that predates by decades, centuries, or millennia the political circumstances of a contemporary event.

## Possible Ways of Approaching the Question

There are several ways in which the question of the relationship of the Israeli state and government to the Jewish people and Judaism in general can be systematically approached. These depend on the various available interpretations of the political, legal, and moral status of Israel itself, each of which opens the way for a specific discourse about the actions of the IDF. If Israel is understood as a contemporary state subject to international law and the legal and moral precepts underlying the United Nations, actions undertaken on its behalf will be subject to assessment and critique in these terms. Specifically, compliance with standards and principles articulated in the U.N. Charter for Human Rights, decisions of the U.N. General Assembly and Security Council and the International Court of Justice (ICJ) will be under examination. This is what is occurring with the ongoing proceedings of the ICJ regarding accusations of genocide and those of other agencies, such as the International Criminal Court (ICC).

If, however, the nature of Israel is understood as the valid embodiment of the aspirations of Zionism—that is, the establishment of a national geographical and political home for people of Jewish background, faith, or identity—the appropriateness and implications of *this* premise will need to be examined. This discussion would focus on whether such an aspiration is itself morally justifiable and what, if any, conditions or limitations may be imposed on its realization. Although this question will not be considered here it is noted that the proposition that Zionism should be identified, or is coterminous, with Judaism has never been widely accepted, even among Jewish people themselves, and today remains highly controversial. In this connection it is relevant to observe that, while the 2018 Israeli “Nation-State” Basic Law commits Israel to being a “national home for the Jewish people,” it makes no reference to Jewish values, philosophy, or culture. Nonetheless, there can be no doubt that, as the “Jewish state,” Israel has since its inception come to be identified as inextricably bound up with Judaism by many Jews and non-Jews.

These considerations lead to a third way in which the question may be considered, which is essentially a philosophical and ethical one. This concerns the nature of Judaism itself, its relationship to the state of Israel, the role of the Israeli government and state apparatus in representing or realizing this relationship, and the criteria and standards according to which policies and actions of the government and army may be assessed and evaluated. This is the approach this essay seeks to take.

There are some problems raised by this last way of formulating the question which need to be acknowledged at the outset. The idea of a “nature of Judaism” is problematic, as would also be the case for attempts to characterize a “nature” of Christianity, Islam, or other religions. However, arguably, the problem is even greater for Judaism than for most other faith-based cultures because of its extremely heterodox character. Indeed, Judaism does not contain a uniquely defined, circumscribed body of ideas or beliefs that could be said to constitute its “essence” and therefore cannot be epitomized in relation to a fixed or clear-cut body of principles or rules. As the philosopher Emmanuel Levinas has expressed:

In the present day, the word Judaism covers several quite distinct concepts. [Nevertheless,] [a]bove all, it designates a religion, [including a] system of beliefs, rituals, and moral prescriptions founded on the Bible, the Talmud and rabbinic literature, and often combined with the mysticism or philosophy of the Kabbalah. (Levinas and Hand 1997)

Additionally, for some, Judaism is a label that describes something much broader than a religion. It describes a culture, an ethnic community, a people, an ethical framework, even a distinct civilization (Kaplan 2010).

In any case, Judaism—like the other religions—is a dynamic field of culture, ideas, beliefs, and moral principles that have evolved over the millennia and remain fluid and contested in the present day. For this reason, it is not possible to explicate formally the precise contents of Judaism in a manner that would allow a direct formal comparison with the statements or actions of a certain group of people. Notwithstanding this, there are claims that can be made, even if sometimes in qualified or conditional terms. While Judaism is a vibrant and evolving culture and its adherents are very diverse, its structures and contents are by no means arbitrary. On the contrary, they have been developed and refined unceasingly over the millennia through an active process of reflection, dialogue and argument which has been rigorously and comprehensively recorded and documented for the generations that follow.^2^

## Available Resources

An immense corpus of Jewish sacred texts and “wisdom literature” has accumulated over the last two and one half millennia. The core of this material takes the form of the Bible (for non-Jews, the “Old Testament”) and other pre-Talmudic texts, the Talmud, and centuries of rabbinic commentaries. It also incorporates a large array of other works in multiple forms that include contemporary reflections and analyses of the core texts.

In seeking to discern the nature and content of a Jewish ethics it is important to acknowledge that the Jewish vision of the world is expressed in the Bible but in the Bible as reflected and interpreted by rabbinic literature, of which the Talmud and its commentaries constitute the leading part. The Talmud, fixed in writing around the sixth century CE, is a compilation of a body of work that forms a tradition that is substantially older than Christianity (Levinas 1997, 30).

The Talmud consists of two main parts: the Mishnah, the written version of oral law, and the Gemara, the discussions and analyses of these laws, supplemented by additional teachings such as the Baraitot (“external writings”). The rabbinic commentaries include a vast array of later interpretations and commentaries, such as the Rashi, the Tosafot, and the writings of sages such as Maimonides (twelfth century) and Rabbi Yosef Karo (sixteenth century), who compiled the most authoritative codification of Jewish law. Subsequent texts have been developed by later generations of scholars who continue to reflect on, expand, and develop the central ideas of these works. This includes a significant contemporary, and constantly expanding, literature (e.g. Neusner 1994; Kraemer 1996; Boyarin 1990; Wimpfheimer 2018).

Together, this large corpus of writings constitutes a resource that provides guidance and stimulates new insights, not only for those deeply steeped in the somewhat arcane traditions of Judaic scholarship but also for individuals in modern society who draw on them, often indirectly and without explicit intention, in their responses to everyday issues and dilemmas. In other words, Judaism is a living, dynamic collection of discourses that has accumulated over millennia and which draws on profound scholarship and life experience. It is this extensive and multifaceted nature that makes it impossible to characterize “Judaism” with a few perfunctory statements. Nevertheless, it also offers firm and clear principles that have emerged, been refined, and have been incorporated in fundamental ways into the patterns of living of those who identify as adherents of Judaism.

As we shall argue, in the Talmud and the rabbinical literature there are some irrefragable, foundational values including but not limited to: respect for life (often referred to as the “sanctity” of life); generosity; a love of peace; opposition to private vengeance; support for justice and mercy; the application of fair processes of reasoning; rejection of collective punishment; minimization of collateral damage and injury to innocent parties; and prohibition of destruction of social and economic infrastructure. These are all articulated and interpreted through the application of an extended, unending process of dialogue, argumentation, and judgment, the outcome of which is an accumulating, and incessantly contested, body of wisdom and knowledge. We argue that, despite this complexity, it is possible to assess and evaluate major ethical challenges in the present day using the resources that make up the Jewish tradition. Indeed, that is their ultimate purpose and intention.

We note that our readings of these texts in this context draw on a particular tradition of scholarship into interpretations of the corpus of traditional Judaic works. While the available hermeneutic strategies span a vast field encompassing many schools and perspectives, the approach we have chosen highlights especially two features we take to be incontestable: the dialogical and polyphonic nature of the texts themselves and the focus on ethical responsibilities, both in face-to-face relationships and in the broader organization of society. Other interpretative strategies emphasize other aspects of the texts, including the logical structure of halakhic (legal) categories, the layers of traditions, the inherent hermeneutic rules, the pilpul method (a dialectical method of Talmudic interpretation popular among Ashkenazi scholars in the sixteenth to eighteenth centuries) etc., but all acknowledge the multifaceted nature of the Talmudic and other rabbinic dialogues as well as their ineluctable preoccupation with resolving the ethical perplexities of daily life (see e.g. Levinas 1979 and 2019; Boyarin 1990, 1993, and 1994).

## Overview of Method

To examine whether the actual acts of the Israeli government and the IDF can be considered to be consistent with the philosophy and ethics of Judaism we will proceed as follows. We will commence by analysing explicit ethical content relating to war and violence, treatment of enemies and civilians, and related issues in the Bible. Specifically, we will seek biblical passages which have been or may be used in public discourse to justify the IDF actions. We do this to emphasize that, in the absence of the refined interpretive lens of the Talmud, it is not difficult to find apparent support in the Bible for extreme acts of violence. Of course, this point has been made many times and it is also well-recognized that there are multiple countervailing perspectives that could also be quoted. However, in view of public pronouncements, by the Israeli government and others, we consider that this is a necessary place to start.

We will then trace how the biblical texts and themes thereby identified are received, interpreted, and endowed with meaning in the Talmud and later literature. We argue that, for all their polysemy, these texts do embody some clear foundational values which they repeatedly apply in their inexorable process of critique and counter-critique. We will then proceed to focus the discussion on specific arguments and potential ethical justifications or criticisms of the IDF actions, seeking to discern Talmudic and later rabbinic views about them. This discussion will seek to draw conclusions about the answer to our key question.

It is emphasized that in the process of argumentation and interpretation we are applying we do not claim that unequivocal and definitive rulings can be provided about the deep and fundamental issues involved. Nor do we in this essay analyse in detail the epistemological and ethical transitions from the Bible to the Talmud, or even within the Talmud from the Mishnah to the Gemara, although we will advert to this important philosophical background. Further, we do highlight the manner in which the texts are characterized by internal tensions and multiple voices, giving rise to interpretive openness and flexibility, and supporting an ongoing dialogical process that deliberately preserves continuing critique and ethical disagreement.

The interpretative framework we have chosen is informed especially by the work of Emmanuel Levinas, for whom the Judaic corpus exemplifies a relational and responsive ethics that assumes and elaborates mutual responsibility mediated via language, and that of Daniel Boyarin, for whom the dialogic, polyphonic, heteroglossic, and culturally situated nature of Talmudic reasoning is a fundamental characteristic (Levinas and Hand 1997; Levinas 1979, 2019; Boyarin 1990, 1993, 1994).

In the second part of this essay we will apply this hermeneutic and methodological perspective to an analysis of key texts in the Jewish ethical tradition. Specifically, we will examine possible justifications for the actions of the Israeli government and the IDF and assess them in relation to the conclusions that can be drawn from this analysis in order to answer the question we have posed.

## Data Availability

Not applicable.

## Footnotes

1 Others include Gisha, Breaking the Silence, Yesh Din, Adalah, Rabbis for Human Rights, Machsom Watch etc. References to the evidence regarding these and other examples of Israeli actions are too numerous to mention and can easily be identified by readers. The references listed provide merely a small sample of the available resources.

2 Cf. Levinas: “Perhaps the ultimate essence of Israel, derives from its innate predisposition to involuntary sacrifice, its exposure to persecution…. to be persecuted, to be guilty without having committed any crime, is not an original sin, but the obverse of a universal responsibility, a responsibility for the other, that is more ancient than any sin” (1997, 225).
